# French crop yield, area and production data for ten staple crops from 1900 to 2018 at county resolution

**DOI:** 10.1038/s41597-022-01145-4

**Published:** 2022-02-03

**Authors:** Bernhard Schauberger, Hiromi Kato, Tomomichi Kato, Daiki Watanabe, Philippe Ciais

**Affiliations:** 1 Potsdam Institute for Climate Impact Research (PIK), Telegrafenberg A31, 14473 Potsdam, Germany; 2 Laboratoire des Sciences du Climat et de l’Environnement, Institut Pierre-Simon Laplace (IPSL), 91191 Gif sur Yvette, France; 3 University of Applied Sciences Weihenstephan-Triesdorf, Department of Sustainable Agriculture and Energy Systems, Freising, Germany; 4 Research Faculty of Agriculture, Hokkaido University, Hokkaido, 060-8589 Sapporo, Japan; 5 Global Station for Food, Land and Water Resources (GSF), Global Institution for Collaborative Research and Education (GI-CoRE), Hokkaido University, Hokkaido, 060-0815 Sapporo, Japan

**Keywords:** Agroecology, Climate-change adaptation

## Abstract

Agricultural performance is influenced by environmental conditions, management decisions and economic circumstances. It is important to quantify their respective contribution to allow for detecting major hazards to production, projecting future yields under climate change and deriving adaptation options. For this purpose, time series of agricultural yields with high spatial and long-term temporal resolution are a primary requisite. Here we present a data set of crop performance in France, one of Europe’s major crop producers. The data set comprises ten crops (barley, maize, oats, potatoes, rapeseed, sugarbeet, sunflower, durum wheat, soft wheat and wine) and covers the years 1900 to 2018. It contains harvested area, production and yield data for all 96 French *départements* (i.e. counties or NUTS3 level) with a total number of 375,264 data points. Entries until 1988 have been digitized manually from statistical yearbooks. The technical validation indicates a high consistency of the data set within itself and with external resources. The data set may contribute to an enhanced understanding of the manifold influences on agricultural performance.

## Abstract (summary)

**Table:** 

Measurement(s)	crop yield • crop area • crop production
Technology Type(s)	digital curation
Sample Characteristic - Environment	agricultural field
Sample Characteristic - Location	Metropolitan France

Machine-accessible metadata file describing the reported data: 10.6084/m9.figshare.17213105

## Background & Summary

Future food provision may be challenged by several factors: climate change, growing global population, shift of dietary patterns, increasing soil degradation and higher pressure on land^1–3^. These strains are already perceived now and their impact on agriculture will likely grow in the future. To better understand and quantify these influences, a comprehensive data base of historical agricultural performance is of salient importance. We present such a data set for France, a major crop producer, with 5%, 2%, 8%, 14%, 4% and 8% of the global production of wheat, maize, barley, sugar beet, sunflower and rapeseed in 2014, respectively.

This paper describes crop performance in France in the full 20^th^ and beginning 21^st^ centuries (1900–2018; 1900–2016 for wine). Ten crops are available on subnational administrative units (*département*, corresponding to counties on NUTS3 (http://ec.europa.eu/eurostat/web/nuts/overview) or GADM2 (http://gadm.org/) levels, with an average area of 5,675 km^2^; henceforth: department). Each entry comprises cultivated area, production and yield data. The crops are barley, maize, oats, potatoes, rapeseed, sugarbeet, sunflower, durum wheat, soft wheat and wine. Four of them (barley, oats, rapeseed and soft wheat) have distinct spring and winter cultivar records, resulting in a total of 18 crop-cultivar types. This unique data set contains a total of 375,264 data points on department level that have been collected and manually digitized (until 1988) over the course of two years from regional statistical offices in France. Yields (in tonnes dry mass, t DM) were calculated from production and area data since the annotations in the statistical year books were often erroneous. All data were subjected to an outlier filtering (see Methods). After filtering, there are 120,942 entries for yields, 127,344 entries for area and 126,978 entries for production. We evaluate data quality internally and by comparison to other established data sources. This data set is a unique resource due to its long-time frame, its high spatial detail and the availability of area, production and yield data.

The data set presented here has been used in two previous studies. The first describes the trends in French yields and discusses possible reasons for recently observed stagnation tendencies^4^, while the second identifies major weather-related hazards for crop production in France^5^. For further discussions about the crop performance data we refer to these studies.

## Methods

### Crop data

Crop area (in hectare, ha, for sown areas) and production (in kg) statistics on departmental level from 1900 until 1988 were collected from books of national agricultural statistics (‘Statistique agricole annuelle’ or ‘Annuaire de statistique agricole’) compiled by the French Ministry of Agriculture; detailed references are provided in the supplementary information. Numbers were manually digitized from photocopied versions of the original paper documents. Data from 1989 to 2018 were derived from digital statistics from the Agreste database (‘Statistique agricole annuelle’ compiled by the Service de la Statistique et de la Prospective (SSP), Secrétariat Général du Ministère de l’Agriculture, de l’Agroalimentaire et de la Forêt (MAAF), France); details are provided in the supplementary information. Yields were calculated from total production and sown area for each department to avoid apparently often incorrect yield values printed in the old statistics books. Yields are given in kilogram per hectare (kg/ha, for sown area) for dry mass with 10–16% moisture content, depending on the crop.

Data are available for ten crops: soft wheat (spring and winter separately), durum wheat, maize, oats (spring and winter), rapeseed (spring and winter), barley (spring and winter), potatoes, sugarbeet, sunflower and wine. The split into spring and winter crops eventually results in 18 distinct crop-cultivar types. Time frames with available data and the correspondence between French and English names are provided in Table 1.

**Table 1:** Data set description for yields on department level.

Crop (*French name*)	Seasonal type	Years with data	Filtered outliers (*fraction of data*)			Number of data points after filtering		
			Yield	Area	Production	Yield	Area	Production
Barley (*Orge*)	Spring	1943–2018	228 (3.7%)	8 (0.1%)	10 (*0.2%*)	5,932	6,805	6,246
	Winter	1943–2018	243 (*3.7%*)	0 (*0%*)	4 (*0.1%*)	6,262	6,563	6,831
	Total	1900–2018	404 (*3.7%*)	9 (*0.1%*)	10 (*0.1%*)	10,381	10,783	10,784
Sugarbeet (*Betterave*)	*(n.a.)*	1900–2018	175 (*3.5%*)	13 (*0.2%*)	16 (*0.3%*)	4,783	5,225	5,103
Maize (*Maïs*)	*(n.a.)*	1900–2018	326 (*3.7%*)	3 (*0.0%*)	8 (*0.1%*)	8,452	8,793	8,784
Oats (*Avoine*)	Spring	1943–2018	232 (*3.7%*)	18 (*0.3%*)	18 (*0.3%*)	6,112	6,376	6,365
	Winter	1943–2018	203 (*3.4%*)	10 (*0.2%*)	14 (*0.2%*)	5,730	5,957	5,952
	Total	1900–2018	424 (*3.9%*)	1 (*0.0%*)	3 (*0.0%*)	10,341	10,781	10,777
Potatoes (*Pommes de terre*)	*(n.a.)*	1900–2018	498 (*4.6%*)	3 (*0.0%*)	52 (*0.5%*)	10,238	10,744	10,690
Rape (*Colza*)	Spring	1943–2018	67 (*2.6%*)	47 (*1.6%*)	14 (*0.5%*)	2,556	2,976	2,826
	Winter	1944–2018	165 (*2.9%*)	1 (*0.0%*)	6 (*0.1%*)	5,469	5,776	6,111
	Total	1900–2018	270 (*3.3%*)	3 (*0.0%*)	9 (*0.1%*)	7,830	8,161	8,219
Sunflower (*Tournesol*)	*(n.a.)*	1943–2018	110 (*3.0%*)	3 (*0.1%*)	5 (*0.1%*)	3,603	3,766	3,734
Soft wheat (*Froment, Blé*)	Spring	1943–2018	167 (*3.3%*)	63 (*1.2%*)	55 (*1.1%*)	4,939	5,110	5,092
	Winter	1943–2018	246 (*3.5%*)	4 (*0.1%*)	1 (*0.0%*)	6,759	7,009	7,010
	Total	1900–2018	378 (*3.5%*)	1 (*0.0%*)	2 (*0.0%*)	10,438	10,822	10,818
Durum wheat (*Blé dur*)	Total	1961–2018	92 (*3.3%*)	5 (*0.2%*)	5 (*0.2%*)	2,682	2,881	2,832
Wine (*Vignoble*)	*(n.a.)*	1900–2016	339 (*3.9%*)	7 (*0.1%*)	45 (*0.5%*)	8,435	8,816	8,804
**Total yield data points**			**4,567 (*3.6%*)**	**199 (*0.2%*)**	**277 (*0.2%*)**	**120,942**	**127,344**	**126,978**

A total of 11,424 data points per crop (96 departments in 119 years) would be possible.

The shapes of French departments have changed over time. We use the 96 mainland (Metropolitan France) departments in their current form and subsume historical values to modern departments as follows. Corsica was one single department until 1975 but then split into Corse-du-Sud and Haute-Corse. Data for Corsica until 1975 were split equally (area, production) or copied (yield) to both new departments. Seine and Seine-et-Oise were two departments until 1967, but then subdivided into seven new departments on 1 January 1968. To account for this, we consider the values of the seven new departments (Essonne, Hauts-de-Seine, Paris, Seine-Saint-Denis, Val-de-Marne, Val-d’Oise, Yvelines) only from 1968 on and unite the two old departments into one counter-factual (“Seine_SeineOise” in the data tables) until 1967.

Multiple cropping per year within this set of crops is accounted for by separate area data, but is practically nonexistent in France^6^.

### Quality filters

Some yield values had to be considered as outliers, also after checking for digitizing errors. There were four criteria for defining an outlier. First, absolute yield values larger than a physiologically currently unreachable threshold were removed; threshold values were 15 t/ha for barley and durum wheat, 200 t/ha for sugarbeet and potatoes, 20 t/ha for maize, oats and wheat, 10 t/ha for rape and sunflower and 200 hl/ha for wine. These thresholds were chosen to eliminate visually obvious outliers likely due to mismatches between area and production records. The values are set slightly above current maximum attained yields, thus remaining permissive and removing only obvious errors in this first step. Additionally, all yield values for winter rape in 1944, spring rape in 1968 and spring barley in 1980 were removed due to wrongly reported values in the yearbooks. This first step removed in total 167 yield data points. Second, the top 1% of yield values across all departments per decade were removed. Third, values above or below the mean +/− four times the standard deviation of each crop-department time series (for yield, area and production separately) were removed. Fourth, and finally, a similar variance filter as in the third step was applied within each decade of a single time series, filtering values above or below decadal mean +/− two (for yield) or three (area, production) decadal standard deviations. The latter three filters removed, on average, 3.6% of the yield and 0.2% of the area or production data, respectively (Table 1). There were, as a median, 43 yield outliers per department (out of 1,260 data points on average), with a range of 4 (department *Hauts de Seine*) and 255 (*Nord*) and an interquartile range of 35–50 outliers. Outliers were masked as missing values to avoid introducing a bias from any correction. In the accompanying data sets we provide two version of the full data set, one without any corrections (“RAW”) and one where the filters described above have been applied (“FILTERED”).

### Validation

Nationally aggregated area, production and yield data from our data set were validated with national data from 1961 to 2018 provided by the FAO (http://faostat3.fao.org/home/E). Area and production data for crops with separate spring and winter data were summed on department level to test agreement with area and production data digitized for the ‘total’ crop.

## Data Records

**Fig. 1:**
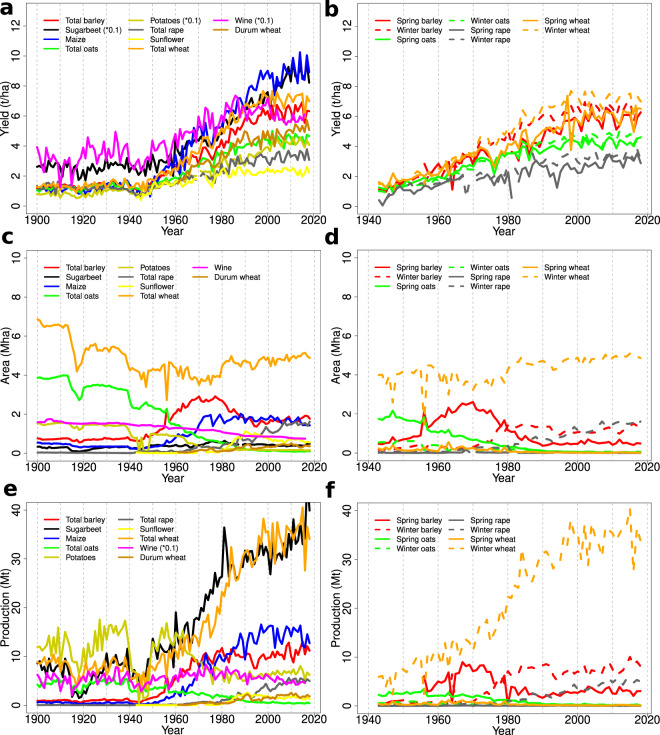
Nationally aggregated yield (**a,b**), area (**c,d**) and production (**e,f**) data. Crops are split by seasonal types for display reasons. Yields for sugarbeet, potatoes and wine (for wine also production) have been scaled with 0.1 for display reasons (indicated in the legends). Yield units are t/ha, area units are hectare (ha) and production units are tons except for wine where these are hl/ha (yields) and hl (production), respectively (both before scaling). Wine data only run from 1900 to 2016.

**Fig. 2:**
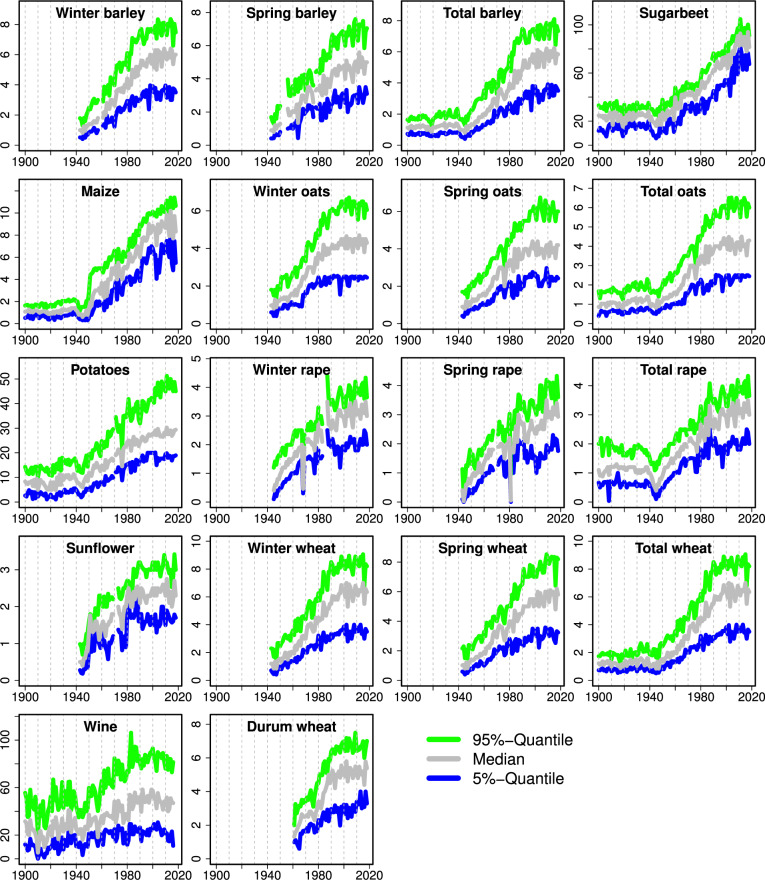
Development of the lowest (blue) and highest 5% (green) percentiles of yields across departments for each year and the range in between (grey). Department yields were aggregated to national level with area weighting. Note the different ranges on the y axis; units are t/ha for all crops except wine where the unit is hl/ha.

Time series length, the number of data points and outlier numbers are provided in Table 1. All results presented afterwards refer only to the filtered data set without outliers. The most complete time series are available for soft wheat, oats, barley, potato, maize and wine. National yield (area-weighted), area and production trends as aggregates over all departments are displayed in Fig. 1. Trends for the bottom and top 5% percentiles as well as the difference between them, i.e. the 90% confidence interval for expected yields, are shown in Fig. 2.

All data described here are available via *GFZ Data Services*, under 10.5880/PIK.2021.001 and with a CC-BY 4.0 license^7^ (see Usage Notes). There are two g-zipped tar balls, one with filtered data (“FILTERED”) and one with unfiltered (“RAW”) data (see Methods). Within each set, the data is organised in tables in plain text files, with one table per crop-cultivar where all three data types (area, production, yield) are combined. This results in 18 tables per filter type. Semicolons (“;”) are used as separators. Diacritic letters of French location names were standardized to the Latin alphabet. Table entries are department name, year of harvest, yield in tonnes/hectare, area in hectare and production in tonnes. Missing values are marked with NA in all three fields. The file name convention is “[crop]_[season-type]_data_1900–2018_[filter-type].txt”; an example filename is”barley_winter_data_1900–2018_FILTERED.txt”. Wine data only cover the years 1900–2016, but follow the same naming convention.

## Technical Validation

Nationally aggregated yield time series were compared with FAO yield data, available from 1961 to 2018. Yields were aggregated from departments with area weighting. For crops with distinct spring and winter types only total yields were compared. Barley, maize, oats, potatoes, rapeseed, sugarbeet, sunflower and soft wheat were available in both data sets; the other crops are not listed by the FAO. All correlation coefficients (Pearson’s *r*) for yield, area and production are at least 0.99, with only five exceptions; all are above 0.95 (Table 2). All correlations are significant with p < 1e-5. These high correlations indicate the subnational data are reasonable. It has to be considered, though, that FAO statistics are compiled from subnational data in France – thus the two data sets are not independent. The high correlations therefore mainly point to the quality of digitalization.

**Table 2:** Correlation of aggregated national time series with FAO data (1961 to 2018).

Crop	Correlation with FAO (Pearson’s r)		
	Area	Production	Yield
Barley	0.999	0.999	0.998
Maize	0.994	0.997	0.998
Oats	0.998	1.000	0.998
Potatoes	0.953	0.968	0.990
Rape	0.999	1.000	0.997
Sugarbeet	0.988	0.999	0.999
Sunflower	0.966	0.994	0.993
Soft wheat	0.978	0.999	0.996

Summed area and production data for crops with separate spring and winter data agree well with area and production data, respectively, for the ‘total’ time series. Pearson’s *r* is at least 0.98 in all cases for area and production, pointing to high consistency in the data. All disagreements are minor and biased to higher area or production values, respectively, when summed from spring and winter data. This may point to some information lacking in the ‘total’ time series, but not on a practically relevant level for national aggregation.

The fraction of outliers, using the criteria defined in the Methods section, was below 4.6% for all crops and below for 4% for most (Table 1). The overall fraction of outliers, which we assume to be annotation errors in the statistical yearbooks, is 3.6% for yields. Outlier numbers for area and production are much lower (0.2%, on average), but in these time series, outlier detection is more difficult since values between departments and years may vary largely without being unreasonable.

Notably, we assume that the values from the early period before World War II are trustworthy in principle, as France has a long tradition (since Napoleon times) of centralized administration with harmonized national directives – also for statistics – in each department. Moreover, the outlier filters did not identify a higher rate of errors during the early period than during later years. Thus, we assume that the area, yield and production data are of sufficient quality to inspect trends and changes in variability also in the early decades of the 20th century.

This data set does not distinguish between rainfed and irrigated yields, which may be a drawback when analyzing, for example, weather influences on crop production. But the area equipped or used for irrigation was not recorded in the handbooks. Statistical methods in the regional statistical offices are not known to have changed over time, such that values can be compared across the complete time frame.

## Usage Notes

The French yield data set described here is available to the general public without any restrictions except citation of this data descriptor paper and the data set^7^ (CC-BY 4.0; Creative Commons License with attribution). The full license text is available with the data set.

In the online repository there are two versions of the data, filtered and unfiltered (see Methods for details). We recommend to use the filtered data only, but have supplied the unfiltered original data, too, to allow for custom filters where appropriate.

Any requests about the data should be directed to one of the corresponding authors. The authors welcome further joint work on the data set.

## Supplementary information

Supplementary Information

## Data Availability

All R (version 3.3.2) codes necessary for analysing the data and producing this data descriptor are publicly available at https://github.com/b-montevideo/French_yields_code. Any requests should be directed to Bernhard Schauberger.
