# Supplementary material for: French crop yield, area and production data for ten staple crops from 1900 to 2018 at county resolution
Source: Sci Data. 2022 Feb 3;9:38. doi: 10.1038/s41597-022-01145-4 (PMC8814188; doi:10.1038/s41597-022-01145-4)
Supplement: Supplementary file 1 — Supplementary Information [file 41597_2022_1145_MOESM1_ESM.pdf]

Data references for Schauberger, Kato et al.: "French crop yield, area and production data for ten staple crops from 1900 to 2018 at county resolution"

Year of data	Author	Title	Issue	Issue Year	Volume	Pages	Original	Location of storage	Serial number / URL	
1900	Ministère de l'agriculture, France	Statistique agricole annuelle 1900	Imprimerie Nationale			118	Book	Université de Caen Basse-Normandie. Bibliothèque universitaire Droit-Lettres	ISSN:1962-5731	
1901	Ministère de l'agriculture, France	Statistique agricole annuelle 1901	Imprimerie Nationale			116	Book	Université de Caen Basse-Normandie. Bibliothèque universitaire Droit-Lettres	ISSN:1962-5731	
1902	Ministère de l'agriculture, France	Statistique agricole annuelle 1902	Imprimerie Nationale			120	Book	Université de Caen Basse-Normandie. Bibliothèque universitaire Droit-Lettres	ISSN:1962-5731	
1903	Ministère de l'agriculture, France	Statistique agricole annuelle 1903	Imprimerie Nationale			108	Book	Université de Caen Basse-Normandie. Bibliothèque universitaire Droit-Lettres	ISSN:1962-5731	
1904	Ministère de l'agriculture, France	Statistique agricole annuelle 1904	Imprimerie Nationale			118	Book	Université de Caen Basse-Normandie. Bibliothèque universitaire Droit-Lettres	ISSN:1962-5731	
1905	Ministère de l'agriculture, France	Statistique agricole annuelle 1905	Imprimerie Nationale			118	Book	Université de Caen Basse-Normandie. Bibliothèque universitaire Droit-Lettres	ISSN:1962-5731	
1906	Ministère de l'agriculture, France	Statistique agricole annuelle 1906	Imprimerie Nationale			311	Book	Bibliothèque nationale de France(BNF)	http://gallica.bnf.fr/ark:/12148/bpt6k284023	
1907	Ministère de l'agriculture, France	Statistique agricole annuelle 1907	Imprimerie Nationale			314	Book	Université de Caen Basse-Normandie. Bibliothèque universitaire Droit-Lettres	ISSN:1962-5731	
1908	Ministère de l'agriculture, France	Statistique agricole annuelle 1908	Imprimerie Nationale			336	Book	Université d'Aix-Marseille. Service commun de la documentation. Bibliothèque universitaire de droit et sciences économiques (Aix-en-Provence. Schuman)	ISSN:1962-5731	
1909	Ministère de l'agriculture, France	Statistique agricole annuelle 1909	Imprimerie Nationale			348	Book	Université de Caen Basse-Normandie. Bibliothèque universitaire Droit-Lettres	ISSN:1962-5731	
1910	Ministère de l'agriculture, France	Statistique agricole annuelle 1910	Imprimerie Nationale			360	Book	Muséum national d'histoire naturelle (Paris). Bibliothèque centrale	ISSN:1962-5731	
1911	Ministère de l'agriculture, France	Statistique agricole annuelle 1911	Imprimerie Nationale			409	Book	Université de Caen Basse-Normandie. Bibliothèque universitaire Droit-Lettres	ISSN:1962-5731	
1912	Ministère de l'agriculture, France	Statistique agricole annuelle 1912	Imprimerie Nationale			420	Book	Université de Caen Basse-Normandie. Bibliothèque universitaire Droit-Lettres	ISSN:1962-5731	
1913	Ministère de l'agriculture, France	Statistique agricole annuelle 1913	Imprimerie Nationale			286	Book	Université d'Aix-Marseille. Service commun de la documentation. Bibliothèque universitaire de droit et sciences économiques (Aix-en-Provence. Schuman)	ISSN:1962-5731	
1914	Ministère de l'agriculture, France	Statistique agricole annuelle 1914	Imprimerie Nationale			330	Book	Conservatoire national des arts et métiers (France). Bibliothèque Centrale (Paris)	ISSN:1962-5731	
1915	Ministère de l'agriculture, France	Statistique agricole annuelle 1915	Imprimerie Nationale			415	Book	Université d'Aix-Marseille. Service commun de la documentation. Bibliothèque universitaire de droit et sciences économiques (Aix-en-Provence. Schuman)	ISSN:1962-5731	
1916	Ministère de l'agriculture, France	Statistique agricole annuelle 1916	Imprimerie Nationale			420	Book	Conservatoire national des arts et métiers (France). Bibliothèque Centrale (Paris)	ISSN:1962-5731	
1917	Ministère de l'agriculture, France	Statistique agricole annuelle 1917	Imprimerie Nationale			420	Book	Université d'Aix-Marseille. Service commun de la documentation. Bibliothèque universitaire de droit et sciences économiques (Aix-en-Provence. Schuman)	ISSN:1962-5731	
1918	Ministère de l'agriculture, France	Statistique agricole annuelle 1918	Imprimerie Nationale			430	Book	Université de Caen Basse-Normandie. Bibliothèque universitaire Droit-Lettres	ISSN:1962-5731	
1919	Ministère de l'agriculture, France	Statistique agricole annuelle 1919	Imprimerie Nationale			452	Book	Conservatoire national des arts et métiers (France). Bibliothèque Centrale (Paris)	ISSN:1962-5731	
1920	Ministère de l'agriculture, France	Statistique agricole annuelle 1920	Imprimerie Nationale			152	Book	Conservatoire national des arts et métiers (France). Bibliothèque Centrale (Paris)	ISSN:1962-5731	
1921	Ministère de l'agriculture, France	Statistique agricole annuelle 1921	Imprimerie Nationale			245	Book	Conservatoire national des arts et métiers (France). Bibliothèque Centrale (Paris)	ISSN:1962-5731	
1922	Ministère de l'agriculture, France	Statistique agricole annuelle 1922	Imprimerie Nationale			164	Book	Conservatoire national des arts et métiers (France). Bibliothèque Centrale (Paris)	ISSN:1962-5731	
1923	Ministère de l'agriculture, France	Statistique agricole annuelle 1923	Imprimerie Nationale			272	Book	Université d'Aix-Marseille. Service commun de la documentation. Bibliothèque universitaire de droit et sciences économiques (Aix-en-Provence. Schuman)	ISSN:1962-5731	
1924	Ministère de l'agriculture, France	Statistique agricole annuelle 1924	Imprimerie Nationale			164	Book	Université d'Aix-Marseille. Service commun de la documentation. Bibliothèque universitaire de droit et sciences économiques (Aix-en-Provence. Schuman)	ISSN:1962-5731	
1925	Ministère de l'agriculture, France	Statistique agricole annuelle 1925	Imprimerie Nationale			274	Book	Conservatoire national des arts et métiers (France). Bibliothèque Centrale (Paris)	ISSN:1962-5731	
1926	Ministère de l'agriculture, France	Statistique agricole annuelle 1926	Imprimerie Nationale			162	Book	Université d'Aix-Marseille. Service commun de la documentation. Bibliothèque universitaire de droit et sciences économiques (Aix-en-Provence. Schuman)	ISSN:1962-5731	
1927	Ministère de l'agriculture, France	Statistique agricole annuelle 1927	Imprimerie Nationale			274	Book	Conservatoire national des arts et métiers (France). Bibliothèque Centrale (Paris)	ISSN:1962-5731	
1928	Ministère de l'agriculture, France	Statistique agricole annuelle 1928	Imprimerie Nationale			162	Book	Conservatoire national des arts et métiers (France). Bibliothèque Centrale (Paris)	ISSN:1962-5731	
1929	Ministère de l'agriculture, France	Statistique agricole annuelle 1929	Imprimerie Nationale			274	Book	Conservatoire national des arts et métiers (France). Bibliothèque Centrale (Paris)	ISSN:1962-5731	
1930	Ministère de l'agriculture, France	Statistique agricole annuelle 1930	Imprimerie Nationale			162	Book	Conservatoire national des arts et métiers (France). Bibliothèque Centrale (Paris)	ISSN:1962-5731	
1931	Ministère de l'agriculture, France	Statistique agricole annuelle 1931	Imprimerie Nationale			274	Book	Insee	ISSN:1962-5731	
1932	Ministère de l'agriculture, France	Statistique agricole annuelle 1932	Imprimerie Nationale			160	Book	Insee	ISSN:1962-5731	
1933	Ministère de l'agriculture, France	Statistique agricole annuelle 1933	Imprimerie Nationale			274	Book	Conservatoire national des arts et métiers (France). Bibliothèque Centrale (Paris)	ISSN:1962-5731	
1934	Ministère de l'agriculture, France	Statistique agricole annuelle 1934	Imprimerie Nationale			160	Book	Conservatoire national des arts et métiers (France). Bibliothèque Centrale (Paris)	ISSN:1962-5731	
1935	Ministère de l'agriculture, France	Statistique agricole annuelle 1935	Imprimerie Nationale			276	Book	Conservatoire national des arts et métiers (France). Bibliothèque Centrale (Paris)	ISSN:1962-5731	
1936	Ministère de l'agriculture, France	Statistique agricole annuelle 1936	Imprimerie Nationale			202	Book	Conservatoire national des arts et métiers (France). Bibliothèque Centrale (Paris)	ISSN:1962-5731	
1937	Ministère de l'agriculture, France	Statistique agricole annuelle 1937	Imprimerie Nationale			348	Book	Conservatoire national des arts et métiers (France). Bibliothèque Centrale (Paris)	ISSN:1962-5731	
1938	Ministère de l'agriculture, France	Statistique agricole annuelle 1938	Imprimerie Nationale			230	Book	Conservatoire national des arts et métiers (France). Bibliothèque Centrale (Paris)	ISSN:1962-5731	
1939	Ministère de l'agriculture, France	Statistique agricole annuelle 1939	Imprimerie Nationale			344	Book	Conservatoire national des arts et métiers (France). Bibliothèque Centrale (Paris)	ISSN:1962-5731	
1940	Ministère de l'agriculture, France	Statistique agricole annuelle 1940	Imprimerie Nationale			267	Book	Bibliothèque interuniversitaire de santé. Pôle pharmacie, biologie et cosmétologie (Paris)	ISSN:1962-5731	
1941	Ministère de l'agriculture, France	Statistique agricole annuelle 1941	Imprimerie Nationale	1943		415	Book	Bibliothèque interuniversitaire de santé. Pôle pharmacie, biologie et cosmétologie (Paris)	ISSN:1962-5731	
1942	Ministère de l'agriculture, France	Statistique agricole annuelle 1942	Imprimerie Nationale			322	Book	Bibliothèque interuniversitaire de santé. Pôle pharmacie, biologie et cosmétologie (Paris)	ISSN:1962-5731	
1943	Ministère de l'agriculture, France	Statistique agricole annuelle 1943	Imprimerie Nationale	1944		479	Book	Bibliothèque interuniversitaire de santé. Pôle pharmacie, biologie et cosmétologie (Paris)	ISSN:1962-5731	
1944	Ministère de l'agriculture, France	Statistique agricole annuelle 1944	Imprimerie Nationale	1944		387	Book	Muséum national d'histoire naturelle (Paris). Bibliothèque centrale	ISSN:1962-5731	
1945	Ministère de l'agriculture, France	Statistique agricole annuelle 1945	Imprimerie Nationale	1947		555	Book	Bibliothèque interuniversitaire de santé. Pôle pharmacie, biologie et cosmétologie (Paris)	ISSN:1962-5731	
1946	Ministère de l'agriculture, France	Statistique agricole annuelle 1946	Imprimerie Nationale	1947		475	Book	Bibliothèque interuniversitaire de santé. Pôle pharmacie, biologie et cosmétologie (Paris)	ISSN:1962-5731	
1947	Ministère de l'agriculture, France	Statistique agricole annuelle 1947	Imprimerie Nationale	1947		580	Book	Bibliothèque interuniversitaire de santé. Pôle pharmacie, biologie et cosmétologie (Paris)	ISSN:1962-5731	
1948	Ministère de l'agriculture, France	Statistique agricole annuelle 1948	Imprimerie Nationale	1949		458	Book	Bibliothèque interuniversitaire de santé. Pôle pharmacie, biologie et cosmétologie (Paris)	ISSN:1962-5731	
1949	Ministère de l'agriculture, France	Statistique agricole annuelle 1949	Imprimerie Nationale	1951		177	Book	Bibliothèque interuniversitaire de santé. Pôle pharmacie, biologie et cosmétologie (Paris)	ISSN:1962-5731	
1950	Ministère de l'agriculture, France	Statistique agricole annuelle 1950	Imprimerie Nationale	1953		183	Book	Bibliothèque interuniversitaire de santé. Pôle pharmacie, biologie et cosmétologie (Paris)	ISSN:1962-5731	
1951	Ministère de l'agriculture, France	Statistique agricole annuelle 1951	Imprimerie Nationale	1953		186	Book	Bibliothèque interuniversitaire de santé. Pôle pharmacie, biologie et cosmétologie (Paris)	ISSN:1962-5731	
1952	Ministère de l'agriculture, France	Statistique agricole annuelle 1952	Imprimerie Nationale	1956		189	Book	Bibliothèque interuniversitaire de santé. Pôle pharmacie, biologie et cosmétologie (Paris)	ISSN:1962-5731	
1953	Ministère de l'agriculture, France	Statistique agricole annuelle 1953	Imprimerie Nationale	1956		189	Book	Bibliothèque interuniversitaire de santé. Pôle pharmacie, biologie et cosmétologie (Paris)	ISSN:1962-5731	
1954	Ministère de l'agriculture, France	Statistique agricole annuelle 1954	Imprimerie Nationale	1957		187	Book	Bibliothèque interuniversitaire de santé. Pôle pharmacie, biologie et cosmétologie (Paris)	ISSN:1962-5731	
1955	Ministère de l'agriculture, France	Statistique agricole annuelle 1955	Imprimerie Nationale	1958		253	Book	Bibliothèque interuniversitaire de santé. Pôle pharmacie, biologie et cosmétologie (Paris)	ISSN:1962-5731	
1956	Ministère de l'agriculture, France	Statistique agricole annuelle 1956	Imprimerie Nationale	1959		277	Book	Bibliothèque interuniversitaire de santé. Pôle pharmacie, biologie et cosmétologie (Paris)	ISSN:1962-5731	
1957	Ministère de l'agriculture, France	Statistique agricole annuelle 1957	Imprimerie Nationale	1960		269	Book	Bibliothèque interuniversitaire de santé. Pôle pharmacie, biologie et cosmétologie (Paris)	ISSN:1962-5731	
1958	Ministère de l'agriculture, France	Statistique agricole 1958	Imprimerie Nationale	1961		279	Book	Bibliothèque interuniversitaire de santé. Pôle pharmacie, biologie et cosmétologie (Paris)	ISSN:0243-6825	
1959	Ministère de l'agriculture, France	Statistique agricole 1959	Imprimerie Nationale	1961		277	Book	Bibliothèque interuniversitaire de santé. Pôle pharmacie, biologie et cosmétologie (Paris)	ISSN:0243-6825	
1960	Ministère de l'agriculture, France	Statistique agricole 1960	Imprimerie Nationale	1962		293	Book	Bibliothèque interuniversitaire de santé. Pôle pharmacie, biologie et cosmétologie (Paris)	ISSN:0243-6825	
1961	Ministère de l'agriculture, France	Statistique agricole 1961	Imprimerie Nationale	1962		439	Book	Bibliothèque interuniversitaire de santé. Pôle pharmacie, biologie et cosmétologie (Paris)	ISSN:0243-6825	
1962	Ministère de l'agriculture, France	Statistique agricole 1962	Imprimerie Nationale	1963		503	Book	Bibliothèque interuniversitaire de santé. Pôle pharmacie, biologie et cosmétologie (Paris)	ISSN:0243-6825	
1963	Ministère de l'agriculture, France	Statistique agricole 1963	Imprimerie Nationale	1964		535	Book	Bibliothèque interuniversitaire de santé. Pôle pharmacie, biologie et cosmétologie (Paris)	ISSN:0243-6825	
1964	Ministère de l'agriculture, France	Statistique agricole 1964	Imprimerie Nationale	1965		533	Book	Bibliothèque interuniversitaire de santé. Pôle pharmacie, biologie et cosmétologie (Paris)	ISSN:0243-6825	
1965	Ministère de l'agriculture, France	Statistique agricole 1965	Imprimerie Nationale	1966		541	Book	Bibliothèque interuniversitaire de santé. Pôle pharmacie, biologie et cosmétologie (Paris)	ISSN:0243-6825	
1966	Ministère de l'agriculture, France	Statistique agricole 1967 résultats de 19	Imprimerie Nationale	1967		543	Book	Bibliothèque interuniversitaire de santé. Pôle pharmacie, biologie et cosmétologie (Paris)	ISSN:0243-6825	
1967	Ministère de l'agriculture, France	Statistique agricole 1968 résultats de 19	Imprimerie Nationale	1968		820	Book	Bibliothèque interuniversitaire de santé. Pôle pharmacie, biologie et cosmétologie (Paris)	ISSN:0243-6825	
1968	Ministère de l'agriculture, France	Statistique agricole 1969 résultats de 19	Imprimerie Nationale	1969	Tome I	366	Book	Bibliothèque interuniversitaire de santé. Pôle pharmacie, biologie et cosmétologie (Paris)	ISSN:0243-6825	
1968	Ministère de l'agriculture, France	Statistique agricole 1969 résultats de 19	Imprimerie Nationale	1969	Tome II	476	Book	Bibliothèque interuniversitaire de santé. Pôle pharmacie, biologie et cosmétologie (Paris)	ISSN:0243-6825	
1969	Ministère de l'agriculture, France	Statistique agricole 1970 résultats de 19	Imprimerie Nationale	1970	Tome I	392	Book	Bibliothèque interuniversitaire de santé. Pôle pharmacie, biologie et cosmétologie (Paris)	ISSN:0243-6825	
1969	Ministère de l'agriculture, France	Statistique agricole 1970 résultats de 19	Imprimerie Nationale	1970	Tome II	471	Book	Bibliothèque interuniversitaire de santé. Pôle pharmacie, biologie et cosmétologie (Paris)	ISSN:0243-6825	
1970	Ministère de l'agriculture, France	Statistique agricole 1971 résultats de 19	Imprimerie Nationale	1971	Tome I	334	Book	Bibliothèque interuniversitaire de santé. Pôle pharmacie, biologie et cosmétologie (Paris)	ISSN:0243-6825	
1970	Ministère de l'agriculture, France	Statistique agricole 1971 résultats de 19	Imprimerie Nationale	1971	Tome II	290	Book	Bibliothèque interuniversitaire de santé. Pôle pharmacie, biologie et cosmétologie (Paris)	ISSN:0243-6825	
1971	Ministère de l'agriculture, France	Statistique agricole 1972 résultats de 19	Imprimerie Nationale	1972	Tome I	418	Book	Bibliothèque interuniversitaire de santé. Pôle pharmacie, biologie et cosmétologie (Paris)	ISSN:0243-6825	
1971	Ministère de l'agriculture, France	Statistique agricole 1972 résultats de 19	Imprimerie Nationale	1972	Tome II	379	Book	Bibliothèque interuniversitaire de santé. Pôle pharmacie, biologie et cosmétologie (Paris)	ISSN:0243-6825	
1972	Ministère de l'agriculture, France	Statistique agricole 1973 résultats de 19	Imprimerie Nationale	1973	Tome I	358	Book	Bibliothèque interuniversitaire de santé. Pôle pharmacie, biologie et cosmétologie (Paris)	ISSN:0243-6825	
1972	Ministère de l'agriculture, France	Statistique agricole 1973 résultats de 19	Imprimerie Nationale	1973	Tome II	438	Book	Bibliothèque interuniversitaire de santé. Pôle pharmacie, biologie et cosmétologie (Paris)	ISSN:0243-6825	
1973	Service central des enquetes et etude	Annuaire de statistique agricole 1974 rés	SCEES	1974	Tome I	362	Book	Bibliothèque interuniversitaire de santé. Pôle pharmacie, biologie et cosmétologie (Paris)	ISSN:0243-6825	
1973	Service central des enquetes et etude	Annuaire de statistique agricole 1974 rés	SCEES	1974	Tome II	503	Book	Bibliothèque interuniversitaire de santé. Pôle pharmacie, biologie et cosmétologie (Paris)	ISSN:0243-6825	
1974	Service central des enquetes et etude	Annuaire de statistique agricole 1975 rés	SCEES	1975	Tome I	366	Book	Bibliothèque interuniversitaire de santé. Pôle pharmacie, biologie et cosmétologie (Paris)	ISSN:0243-6825	
1974	Service central des enquetes et etude	Annuaire de statistique agricole 1975 rés	SCEES	1975	Tome II	507	Book	Bibliothèque interuniversitaire de santé. Pôle pharmacie, biologie et cosmétologie (Paris)	ISSN:0243-6825	
1975	Service central des enquetes et etude	Annuaire de statistique agricole 1976 rés	SCEES	1976	Tome I	386	Book	Bibliothèque interuniversitaire de santé. Pôle pharmacie, biologie et cosmétologie (Paris)	ISSN:0243-6825	
1975	Service central des enquetes et etude	Annuaire de statistique agricole 1976 rés	SCEES	1976	Tome II	499	Book	Institut national d'études démographiques (Paris). Service de documentation	ISSN:0243-6825	
1976	Service central des enquetes et etude	Annuaire de statistique agricole 1977 rés	SCEES	1977	Tome I	386	Book	Bibliothèque interuniversitaire de santé. Pôle pharmacie, biologie et cosmétologie (Paris)	ISSN:0243-6825	
1976	Service central des enquetes et etude	Annuaire de statistique agricole 1977 rés	SCEES	1977	Tome II	513	Book	Insee	ISSN:0243-6825	
1977	Service central des enquetes et etude	Annuaire de statistique agricole 1978 rés	SCEES	1978	Tome I	416	Book	Insee	ISSN:0243-6825	
1977	Service central des enquetes et etude	Annuaire de statistique agricole 1978 rés	SCEES	1978	Tome II	521	Book	Institut national d'études démographiques (Paris). Service de documentation	ISSN:0243-6825	
1978	Service central des enquetes et etude	Annuaire de statistique agricole 1979 rés	SCEES	1979	Tome I	406	Book	Institut national d'études démographiques (Paris). Service de documentation	ISSN:0243-6825	
1978	Service central des enquetes et etude	Annuaire de statistique agricole 1979 rés	SCEES	1979	Tome II	521	Book	Institut national d'études démographiques (Paris). Service de documentation	ISSN:0243-6825	
1979	Service central des enquetes et etude	Annuaire de statistique agricole 1980 rés	SCEES	1980	Tome I	410	Book	Insee	ISSN:0243-6825	
1979	Service central des enquetes et etude	Annuaire de statistique agricole 1980 rés	SCEES	1980	Tome II	521	Book	Institut national d'études démographiques (Paris). Service de documentation	ISSN:0243-6825	
1980	Service central des enquetes et etude	Annuaire de statistique agricole 1981 rés	SCEES	1981	Tome I	420	Book	Institut national d'études démographiques (Paris). Service de documentation	ISSN:0243-6825	
1980	Service central des enquetes et etude	Annuaire de statistique agricole 1981 rés	SCEES	1981	Tome II	525	Book	Institut national d'études démographiques (Paris). Service de documentation	ISSN:0243-6825	
1981	Service central des enquetes et etude	Annuaire de statistique agricole 1982 rés	SCEES	1982	Tome I	301	Book	Institut national d'études démographiques (Paris). Service de documentation	ISSN:0243-6825	
1981	Service central des enquetes et etude	Annuaire de statistique agricole 1982 rés	SCEES	1982	Tome II	143	Book	Institut national d'études démographiques (Paris). Service de documentation	ISSN:0243-6825	
1982	Service central des enquetes et etude	Annuaire de statistique agricole 1983 rés	SCEES	1983	Tome I	292	Book	Institut national d'études démographiques (Paris). Service de documentation	ISSN:0243-6825	
1982	Service central des enquetes et etude	Annuaire de statistique agricole 1983 rés	SCEES	1983	Tome II	143	Book	Insee	ISSN:0243-6825	
1983	Service central des enquetes et etude	Annuaire de statistique agricole 1984 rés	SCEES	1984	Tome I	289	Book	Institut national d'études démographiques (Paris). Service de documentation	ISSN:0243-6825	
1983	Service central des enquetes et etude	Annuaire de statistique agricole 1984 rés	SCEES	1984	Tome II	143	Book	Institut national d'études démographiques (Paris). Service de documentation	ISSN:0243-6825	
1984	Service central des enquetes et etude	Annuaire de statistique agricole 1985 rés	SCEES	1985	Tome I	292	Book	Institut national d'études démographiques (Paris). Service de documentation	ISSN:0243-6825	
1984	Service central des enquetes et etude	Annuaire de statistique agricole 1985 rés	SCEES	1985	Tome II	145	Book	Institut national d'études démographiques (Paris). Service de documentation	ISSN:0243-6825	
1985	Service central des enquetes et etude	Annuaire de statistique agricole 1986 rés	SCEES	1986		214	Book	Institut national d'études démographiques (Paris). Service de documentation	ISSN:0243-6825	
1986	Service central des enquetes et etude	Annuaire de statistique agricole 1987 rés	SCEES	1987		458	Book	Institut national d'études démographiques (Paris). Service de documentation	ISSN:0243-6825	
1987	Service central des enquetes et etudes statistiques, Ministere de l'agriculture, F		SCEES	1988			Book		ISSN:0243-6825	
1988	Service central des enquetes et etude	Statistique agricole annuelle résultats dé	SCEES	1989		156	Book		ISSN:0243-6825	
1989	Service de la statistique et de la pros	Statistique agricole annuelle (SAA)	Agreste				Table	Agreste; statistique agricole annuelle	https://agreste.agriculture.gouv.fr/agreste-web/meth	Available on demand at fatima.bouhaddi@agriculture.gouv.fr or angelique.toulon@agriculture.gouv.fr
1990	Service de la statistique et de la pros	Statistique agricole annuelle (SAA)	Agreste				Table	Agreste; statistique agricole annuelle	https://agreste.agriculture.gouv.fr/agreste-web/meth	Available on demand at fatima.bouhaddi@agriculture.gouv.fr or angelique.toulon@agriculture.gouv.fr
1991	Service de la statistique et de la pros	Statistique agricole annuelle (SAA)	Agreste				Table	Agreste; statistique agricole annuelle	https://agreste.agriculture.gouv.fr/agreste-web/meth	Available on demand at fatima.bouhaddi@agriculture.gouv.fr or angelique.toulon@agriculture.gouv.fr

1992	Service de la statistique et de la prosp	Statistique agricole annuelle (SAA)	Agreste	Table	Agreste; statistique agricole annuelle	https://agreste.agriculture.gouv.fr/agreste-web/meth	Available on demand at fatima.bouhaddi@agriculture.gouv.fr or angelique.toulon@agriculture.gouv.fr
1993	Service de la statistique et de la prosp	Statistique agricole annuelle (SAA)	Agreste	Table	Agreste; statistique agricole annuelle	https://agreste.agriculture.gouv.fr/agreste-web/meth	Available on demand at fatima.bouhaddi@agriculture.gouv.fr or angelique.toulon@agriculture.gouv.fr
1994	Service de la statistique et de la prosp	Statistique agricole annuelle (SAA)	Agreste	Table	Agreste; statistique agricole annuelle	https://agreste.agriculture.gouv.fr/agreste-web/meth	Available on demand at fatima.bouhaddi@agriculture.gouv.fr or angelique.toulon@agriculture.gouv.fr
1995	Service de la statistique et de la prosp	Statistique agricole annuelle (SAA)	Agreste	Table	Agreste; statistique agricole annuelle	https://agreste.agriculture.gouv.fr/agreste-web/meth	Available on demand at fatima.bouhaddi@agriculture.gouv.fr or angelique.toulon@agriculture.gouv.fr
1996	Service de la statistique et de la prosp	Statistique agricole annuelle (SAA)	Agreste	Table	Agreste; statistique agricole annuelle	https://agreste.agriculture.gouv.fr/agreste-web/meth	Available on demand at fatima.bouhaddi@agriculture.gouv.fr or angelique.toulon@agriculture.gouv.fr
1997	Service de la statistique et de la prosp	Statistique agricole annuelle (SAA)	Agreste	Table	Agreste; statistique agricole annuelle	https://agreste.agriculture.gouv.fr/agreste-web/meth	Available on demand at fatima.bouhaddi@agriculture.gouv.fr or angelique.toulon@agriculture.gouv.fr
1998	Service de la statistique et de la prosp	Statistique agricole annuelle (SAA)	Agreste	Table	Agreste; statistique agricole annuelle	https://agreste.agriculture.gouv.fr/agreste-web/meth	Available on demand at fatima.bouhaddi@agriculture.gouv.fr or angelique.toulon@agriculture.gouv.fr
1999	Service de la statistique et de la prosp	Statistique agricole annuelle (SAA)	Agreste	Table	Agreste; statistique agricole annuelle	https://agreste.agriculture.gouv.fr/agreste-web/meth	Available on demand at fatima.bouhaddi@agriculture.gouv.fr or angelique.toulon@agriculture.gouv.fr
2000	Service de la statistique et de la prosp	Statistique agricole annuelle (SAA)	Agreste	Table	Agreste; statistique agricole annuelle	https://agreste.agriculture.gouv.fr/agreste-saiku/?plugin=true&query=query/open/SAANR_DEVELOPPE_2#query/open/SAANR_DEVELOPPE_2	
2001	Service de la statistique et de la prosp	Statistique agricole annuelle (SAA)	Agreste	Table	Agreste; statistique agricole annuelle	https://agreste.agriculture.gouv.fr/agreste-saiku/?plugin=true&query=query/open/SAANR_DEVELOPPE_2#query/open/SAANR_DEVELOPPE_2	
2002	Service de la statistique et de la prosp	Statistique agricole annuelle (SAA)	Agreste	Table	Agreste; statistique agricole annuelle	https://agreste.agriculture.gouv.fr/agreste-saiku/?plugin=true&query=query/open/SAANR_DEVELOPPE_2#query/open/SAANR_DEVELOPPE_2	
2003	Service de la statistique et de la prosp	Statistique agricole annuelle (SAA)	Agreste	Table	Agreste; statistique agricole annuelle	https://agreste.agriculture.gouv.fr/agreste-saiku/?plugin=true&query=query/open/SAANR_DEVELOPPE_2#query/open/SAANR_DEVELOPPE_2	
2004	Service de la statistique et de la prosp	Statistique agricole annuelle (SAA)	Agreste	Table	Agreste; statistique agricole annuelle	https://agreste.agriculture.gouv.fr/agreste-saiku/?plugin=true&query=query/open/SAANR_DEVELOPPE_2#query/open/SAANR_DEVELOPPE_2	
2005	Service de la statistique et de la prosp	Statistique agricole annuelle (SAA)	Agreste	Table	Agreste; statistique agricole annuelle	https://agreste.agriculture.gouv.fr/agreste-saiku/?plugin=true&query=query/open/SAANR_DEVELOPPE_2#query/open/SAANR_DEVELOPPE_2	
2006	Service de la statistique et de la prosp	Statistique agricole annuelle (SAA)	Agreste	Table	Agreste; statistique agricole annuelle	https://agreste.agriculture.gouv.fr/agreste-saiku/?plugin=true&query=query/open/SAANR_DEVELOPPE_2#query/open/SAANR_DEVELOPPE_2	
2007	Service de la statistique et de la prosp	Statistique agricole annuelle (SAA)	Agreste	Table	Agreste; statistique agricole annuelle	https://agreste.agriculture.gouv.fr/agreste-saiku/?plugin=true&query=query/open/SAANR_DEVELOPPE_2#query/open/SAANR_DEVELOPPE_2	
2008	Service de la statistique et de la prosp	Statistique agricole annuelle (SAA)	Agreste	Table	Agreste; statistique agricole annuelle	https://agreste.agriculture.gouv.fr/agreste-saiku/?plugin=true&query=query/open/SAANR_DEVELOPPE_2#query/open/SAANR_DEVELOPPE_2	
2009	Service de la statistique et de la prosp	Statistique agricole annuelle (SAA)	Agreste	Table	Agreste; statistique agricole annuelle	https://agreste.agriculture.gouv.fr/agreste-saiku/?plugin=true&query=query/open/SAANR_DEVELOPPE_2#query/open/SAANR_DEVELOPPE_2	
2010	Service de la statistique et de la prosp	Statistique agricole annuelle (SAA)	Agreste	Table	Agreste; statistique agricole annuelle	https://agreste.agriculture.gouv.fr/agreste-saiku/?plugin=true&query=query/open/SAANR_DEVELOPPE_2#query/open/SAANR_DEVELOPPE_2	
2011	Service de la statistique et de la prosp	Statistique agricole annuelle (SAA)	Agreste	Table	Agreste; statistique agricole annuelle	https://agreste.agriculture.gouv.fr/agreste-saiku/?plugin=true&query=query/open/SAANR_DEVELOPPE_2#query/open/SAANR_DEVELOPPE_3	
2012	Service de la statistique et de la prosp	Statistique agricole annuelle (SAA)	Agreste	Table	Agreste; statistique agricole annuelle	https://agreste.agriculture.gouv.fr/agreste-saiku/?plugin=true&query=query/open/SAANR_DEVELOPPE_2#query/open/SAANR_DEVELOPPE_4	
2013	Service de la statistique et de la prosp	Statistique agricole annuelle (SAA)	Agreste	Table	Agreste; statistique agricole annuelle	https://agreste.agriculture.gouv.fr/agreste-saiku/?plugin=true&query=query/open/SAANR_DEVELOPPE_2#query/open/SAANR_DEVELOPPE_5	
2014	Service de la statistique et de la prosp	Statistique agricole annuelle (SAA)	Agreste	Table	Agreste; statistique agricole annuelle	https://agreste.agriculture.gouv.fr/agreste-saiku/?plugin=true&query=query/open/SAANR_DEVELOPPE_2#query/open/SAANR_DEVELOPPE_6	
2015	Service de la statistique et de la prosp	Statistique agricole annuelle (SAA)	Agreste	Table	Agreste; statistique agricole annuelle	https://agreste.agriculture.gouv.fr/agreste-saiku/?plugin=true&query=query/open/SAANR_DEVELOPPE_2#query/open/SAANR_DEVELOPPE_7	
2016	Service de la statistique et de la prosp	Statistique agricole annuelle (SAA)	Agreste	Table	Agreste; statistique agricole annuelle	https://agreste.agriculture.gouv.fr/agreste-saiku/?plugin=true&query=query/open/SAANR_DEVELOPPE_2#query/open/SAANR_DEVELOPPE_8	
2017	Service de la statistique et de la prosp	Statistique agricole annuelle (SAA)	Agreste	Table	Agreste; statistique agricole annuelle	https://agreste.agriculture.gouv.fr/agreste-saiku/?plugin=true&query=query/open/SAANR_DEVELOPPE_2#query/open/SAANR_DEVELOPPE_9	
2018	Service de la statistique et de la prosp	Statistique agricole annuelle (SAA)	Agreste	Table	Agreste; statistique agricole annuelle	https://agreste.agriculture.gouv.fr/agreste-saiku/?plugin=true&query=query/open/SAANR_DEVELOPPE_2#query/open/SAANR_DEVELOPPE_10	
